# 1215. Variation in Hand Hygiene Improvement After Implementation of an Electronic Hand Hygiene System During the COVID-19 Pandemic

**DOI:** 10.1093/ofid/ofac492.1047

**Published:** 2022-12-15

**Authors:** James P Steinberg, Elizabeth Overton, Nancye Feistritzer, Kari L Love, Jill Holdsworth, Margaret Whitson`, Lorry Lewis, Lorry Lewis, Julie Swann, Jesse T Jacob

**Affiliations:** Emory University School of Medicine, Atlanta, Georgia; Emory Healthcare, Atlanta, Georgia; Emory Healthcare, Atlanta, Georgia; Emory Healthcare, Atlanta, Georgia; Emory University Hospital Midtown, Atlanta, Georgia; Emory Healthcare, Atlanta, Georgia; Emory Healthcare, Atlanta, Georgia; Emory Healthcare, Atlanta, Georgia; Emory Healthcare, Atlanta, Georgia; Emory University School of Medicine, Atlanta, GA; Georgia Emerging Infections Program, Atlanta, GA, Atlanta, Georgia

## Abstract

**Background:**

Electronic hand hygiene (HH) monitoring systems have many potential advantages but there are limited data on wide-scale implementation of these systems.

**Table:**
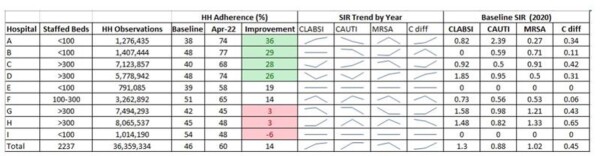
Hand Hygiene Observations, Improvement in Adherence Rates and SIR Trends by Hospital

**Methods:**

We deployed an electronic HH monitoring system in over 2,100 acute and critical care rooms across 9 hospitals in an academic health system. Badges with a Bluetooth beacon were issued to over 7,000 healthcare workers. Deployment began in early 2020 and was interrupted by the pandemic. The rollout of interventions to improve HH adherence was managed at the hospital level. Healthcare-associated infections (HAIs) were determined by the infection prevention team using standard CDC definitions. Hospital-level HH adherence rates were compared to a composite SIR including SIRs for CLABSI, CAUTI, hospital-onset MRSA bloodstream infections and hospital-onset Clostridiodes difficile infections.

**Results:**

Between January 2020 and April 2022, there were over 36 million hand hygiene opportunities with an average of 19 observations per staffed room per day. Overall HH adherence improved from 46% to 60%, with significant variation by hospital (4 improving by >25% and 3 by < 5%). Hospitals whose implementation was most delayed showed the least improvement. Preliminary analysis found no relationship between hand hygiene improvement and the SIR composite aggregated by calendar year.

**Conclusion:**

Despite the challenges of large-scale implementation of an electronic HH system during a pandemic, we demonstrated an overall improvement in HH adherence. The wide variation in improvement among hospitals was due to timing of implementation, variation in the dedicated hospital-specific project management resources and leadership engagement. In addition to technology, successful implementation of electronic HH systems requires dedicated resources and culture change. Pandemic-related staffing challenges, disruption of standard HAI prevention efforts and intensive device utilization confounded our ability to show a relationship between HH adherence and HAI rates.

**Disclosures:**

**All Authors**: No reported disclosures.

